# Comparative characterization of organ-specific phase I and II biotransformation enzyme kinetics in salmonid S9 sub-cellular fractions and cell lines

**DOI:** 10.1007/s10565-025-09992-8

**Published:** 2025-01-28

**Authors:** Baptiste P. M. Martin, Marco E. Franco, Kristin Schirmer

**Affiliations:** 1 Department of Environmental Toxicology, Swiss Federal Institute of Aquatic Science and Technology, Eawag, 8600 Dübendorf, Switzerland; 2 Department of Environmental Systems Science, ETH Zürich, 8092 Zürich, Switzerland

**Keywords:** Chemical pollution, Chemical transformation and elimination, Fish, In vitro, Rainbow trout (*Oncorhynchus mykiss*), Brown trout (*Salmo trutta*)

## Abstract

Advancing in vitro systems to address the effects of chemical pollution requires a thorough characterization of their functionalities, such as their repertoire of biotransformation enzymes. Currently, knowledge regarding the presence, activity magnitudes, and inducibility of different biotransformation pathways in vitro is scarce, particularly across organs. We report organ-specific kinetics for phase I and II biotransformation enzymes, under basal and induced conditions, in two in vitro systems using salmonid fish: S9 sub-cellular fractions from brown trout (*Salmo trutta*) and rainbow trout (*Oncorhynchus mykiss*) were compared with rainbow trout cell lines. Cyp1a and glutathione S-transferase (Gst) activities were the highest in liver S9 fractions and RTL-W1 liver cells, yet systems derived from the intestine, gills, and brain also displayed these biotransformation pathways. Cyp3a-like activity was only measurable in liver and intestinal S9 fractions, but all rainbow trout cell lines, including RTgill-W1 and RTbrain, displayed this type of activity. Furthermore, despite RTgutGC having the highest constitutive Cyp3a-like activity, its inducibility was the highest in RTL-W1 cells. Similarly, both RTL-W1 and RTgutGC cells displayed Cyp2b-like activity, but this was only measurable upon induction. Contrarily, S9 fractions from the liver, intestine and gills displayed constitutive Cyp2b-like activity. While these differences could be related to differential functionality of biological processes at the in vivo level, we provide important evidence of a broad spectrum of in vitro enzymatic activity in salmonid models. As such, both S9 fractions and cell lines represent important alternatives to animal testing for evaluating the biotransformation and bioaccumulation of environmental pollutants.

**Graphical Abstract:**

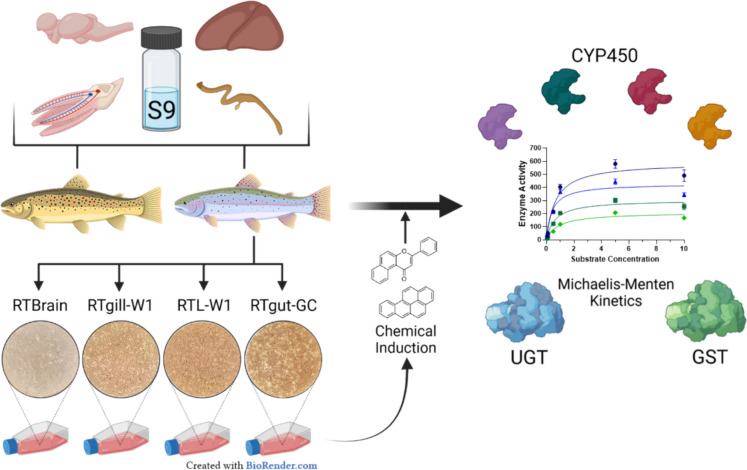

**Supplementary Information:**

The online version contains supplementary material available at 10.1007/s10565-025-09992-8.

## Introduction

Aquatic organisms, including salmonids, are equipped with an array of processes that allow them to respond to chemical pollution. Among these responses, those associated with the chemical defensome (Eide et al. 2021; Goldstone et al. 2006) are of particular interest to further understand how chemicals alter the integrity of organisms but also how organisms may cope with exposure. For example, biotransformation processes have been recognized as major components of the chemical defensome in environmental toxicology since these processes represent an important facet when assessing the adverse effects of pollution (see reviews by Kleinow et al. (1987) and Schlenk et al. (2024)). Indeed, biotransformation processes are critical in many detoxification pathways and in reducing the bioaccumulation potential of chemicals (Schlenk et al. 2024).

Fish have been the focus of many efforts aiming to investigate the role of the chemical defensome in maintaining the integrity of aquatic species inhabiting polluted environments. For ethical, time, financial, and legal reasons, but also to obtain transferable, mechanistic knowledge efficiently, there is a well-recognized demand for cell-based and other in vitro testing strategies in particular for evaluating biotransformation pathways (Franco and Lavado 2019; Nichols et al. 2018). These strategies often entail the use of sub-cellular preparations, such as S9 fractions, which correspond to the cytosolic/enzymatic fractions resulting from the lysis of e.g. hepatocytes (Johanning et al. 2012), and cell lines, which maintain their membrane integrity and production of endogenous co-factors to support enzymatic activity without further animal use (Fent 2001; Rehberger et al. 2018; Segner 2015).

For example, the role of Cyp1a isoforms as major contributors to the biotransformation capacity of different fish species has been extensively and successfully assessed both in vivo and in vitro*,* despite differences in magnitude across tissues, species, and experimental models (Bols et al. 2005; Franco et al. 2024). Assessments of Cyp1a have included evaluations of inducibility (Behrens et al. 2001; Billiard et al. 2004; Ferreira et al. 2014; Ortiz-Delgado et al. 2008; Santos et al. 2018; Scholz and Segner 1999; Traven et al. 2008), inhibition (Dasgupta and McElroy 2017; Willett et al. 2001; Wills et al. 2009), tissue-specific responses (Burkina et al. 2021; Santos and dos Reis Martinez 2020), and clearance of e.g. legacy contaminants, like polycyclic aromatic hydrocarbons (PAHs) (Franco et al. 2022b; Stadnicka-Michalak et al. 2018). Similarly, the glutathione S-transferase (Gst) family of enzymes, a major player of phase II biotransformation processes, has been described in fish and fish cell lines by using chemical substrates such as phenobarbital, 2,3,7,8-tetrachlorodibenzo-*p*-dioxin (TCDD), and 1-chloro-2,4-dinitrobenzene (CDNB) (Petřivalský et al. 2009; Tierbach et al. 2020, 2018).

Other studies have elucidated the presence and activity of additional biotransformation pathways that are of relevance when assessing exposure to diverse, common pollutants, such as halogenated aromatic hydrocarbons, pharmaceuticals, pesticides, and personal care products. For example, Cyp2b induction has been observed following phenobarbital exposure, and while the Cyp2b ortholog is absent in many teleost fish (Iwata et al. 2002), significant Cyp2b-like activity has been described in microsomes from killifish (*Fundulus heteroclitus*) and rainbow trout (*Oncorhynchus mykiss*) (Smith and Wilson 2010). Similarly, Cyp3a-associated activity has been reported across organs when using rainbow trout S9 fractions (Burkina et al. 2021) and in hepatic cell lines derived from the rainbow trout, zebrafish (*Danio rerio*), topminnow (*Poecilipsis lucida*), and the Japanese eel (*Anguilla japonica*) (Christen et al. 2009; Creusot et al. 2015; Franco et al. 2018; Thibaut et al. 2009).

Despite these scientific advances, systematic investigations of the biotransformation portfolio of S9 fractions and cell lines, in particular for the evaluation of substrate specificities, activity magnitudes, and inducibility across organs and species is largely lacking. For example, standardized methodologies for assessing biotransformation in fish, like the OECD Test Guidelines 319a and b (OECD 2018b, c), focus on the liver as the organ with the highest biotransformation potential for xenobiotics and endogenous compounds. Only a few studies have explored organ-specific biotransformation activity and its contribution to the clearance of different pollutants using sub-cellular fractions (Franco et al. 2024; Saunders et al. 2020) and cell-based models (Stadnicka-Michalak et al. 2018). This lack of knowledge leads to skepticism as to the biotransformation capabilities of in vitro systems and their application in environmental risk assessment schemes aiming to reduce reliance on animal experimentation.

Therefore, in the present study, we compared biotransformation enzyme kinetics in S9 sub-cellular fractions isolated from the liver, intestine, gills, and brain of two salmonid species: the brown trout (*Salmo trutta;* BT-S9) and rainbow trout (*Oncorhynchus mykiss;* RT-S9). Then, we explored the behavior of biotransformation pathways between the RT-S9 and the rainbow trout cell lines RTL-W1, RTgutGC, RTgill-W1, and RTbrain. In both cases, we focused on the kinetics of major phase I and II biotransformation enzymes, including Cyp1a, Cyp2b, and Cyp3a, as well as glutathione S-transferase (Gst) and UDP-glucuronosyltransferase (Ugt), under basal and induced conditions. This selection was based on the fundamental role of these enzyme pathways in the processing of environmental pollutants in fish (Franco and Lavado 2019; Iwata et al. 2002; Ladd et al. 2016; Luo et al. 2004; Schlenk et al. 2024; Tierbach et al. 2018). The observations from this study aim to advance the knowledge about organ-specific presence and kinetics of different biotransformation enzymes using in vitro systems with fish.

## Materials and methods

### Chemicals and reagents

The enzyme substrates 7-ethoxyresorufin (7-ER, > 95%), 7-pentoxyresorufin (7-PR, > 95%), and 7-benzyloxy-4-(trifluoromethyl)coumarin (BFC, > 97%), and the standard 7-Hydroxy-4-(trifluoromethyl)coumarin (> 95%) were obtained from Chemodex (St. Gallen, CH). Resorufin sodium salt, fluorescein, fluorescamine (> 98%), 1-Chloro-2,3-dinitrobenzene (> 99%), *p*-nitrophenol (> 99%), and the enzyme co-factors reduced β-nicotinamide adenine dinucleotide 2’-phosphate, tetrasodium salt (NADPH; 96%), L-Glutathione reduced (GSH, > 98%), and uridine 5’-diphosphoglucuronic acid trisodium salt (UDPGA, > 98%) were purchased from Merck (Darmstadt, DE). Bovine serum albumin (BSA, > 98%) and Triton X-100 were from Sigma and also purchased from Merck (Darmstadt, DE). Dibenzylfluorescein (> 98%) was purchased from Santa Cruz Biotechnology (Huissen, NL). Alamethicin (> 96%) from *Trichoderma viride* was obtained from AdipoGen (Liestal, CH).

### Experimental models

#### S9 sub-cellular fractions

S9 sub-cellular fractions were isolated from the liver, intestine, gills, and brain of two salmonid species obtained from the Bremgarten fish farm (www.fischzucht-bremgarten.ch) in Switzerland. Brown trout (*Salmo trutta,* n = 9) were grouped into 3 pools of 3 individuals each, while rainbow trout (*Oncorhynchus mykiss,* n = 12) were grouped into 3 pools of 4 individuals each. Fish were euthanized by electric shock following the farm’s in-house protocols, measured for basic morphometric parameters, and dissected on-site. Average length (cm), weight (g), condition factor (*K*), and fractional organ weights (%) from the two species used in S9 fraction preparations are reported in Table S1 and in Franco et al. (2024).

Upon dissection, the liver was perfused through the hepatic vein, excised, and weighed. Livers from each fish pool were then minced and placed in two volumes of ice-cold homogenization buffer following standard protocols (Johanning et al. 2012; OECD 2018c). Intestinal S9 fractions were prepared from collecting the intestinal mucosa in the anterior intestine, an area with high enterocyte density, and following the protocols described in McElroy and Kleinow (1992) and Saunders et al. (2020). Similarly, the preparation of S9 fractions from the gills was based on the protocol by Gomez et al. (2010), with additional clean-up procedures for blood removal. Clean arches were minced into 2–3 mm pieces and pooled into conical tubes with two volumes of chilled homogenization buffer. Lastly, brain S9 sub-cellular fractions were isolated from whole brains. Brains were removed from the cranial cavity and pooled into 15 mL conical tubes containing two volumes of homogenization buffer.

All organ suspensions were further homogenized on ice using a digital ULTRA-TURRAX® homogenizer (IKA®-Werke GmbH & Co. KG, Staufen, DE), and centrifuged at high-speed (13,000 g for 25 min at 4 °C; UniCn MR, Herolab GmbH, Wiesloch, DE). The supernatants were then collected, transferred to cryogenic vials, and stored at −80 °C until assays were performed. Total protein content in the S9 fractions was determined with the Coomassie blue assay (Bradford 1976), using bovine serum albumin (BSA) as a standard. Specific details about solutions and procedures used in S9 fraction preparation are provided as supplemental information (*SI; Sect. 1.1*).

#### Cell lines

Rainbow trout cell lines from the liver (RTL-W1, passages 75—111), intestine (RTgutGC, passages 51—61), gills (RTgill-W1, passages 79—86) and brain (RTbrain, passages 26—34) were routinely cultured at 19 ± 1 °C using L-15 cell culture medium (Leibovitz’s, PAN biotech), supplemented with 5% (10% for RTbrain) of fetal calf serum (FCS; Eurobio Scientific). Regular microscopic inspections were conducted to monitor the growth and quality of the cell monolayers.

All enzyme activity bioassays were conducted in transparent, flat-bottom, 96-well plates for cell culture and with a minimum of two biological replicates with six technical replicates each. Once cells reached confluence, they were transferred from culture flasks to the 96-well plates at densities of 20,000 cells per well (RTL-W1 and RTgutGC) or 40,000 cells per well (RTgill-W1 and RTbrain) in 200 µL. Seeded cells were incubated for 24 h prior to conducting bioassays.

### Enzyme kinetics

Phase I and II biotransformation enzymes were evaluated on whether they displayed differences in Michaelis–Menten kinetics. Michaelis–Menten kinetics allow for the evaluation of enzymatic activity at different substrate concentrations and the derivation of rate constants (*Km*), as a measure of substrate affinity, and maximum reaction rates (*V*max) when enzymes reach a level of substrate saturation (Cornish-Bowden 2015). These parameters were used as indicators of the enzyme-specific biotransformation capacity between different organs and test systems.

#### Constitutive (basal) activity

Many biotransformation enzymes in vertebrates are constantly expressed across different tissues as they are responsible for a range of key endogenous functions (see reviews by Arellano-Aguilar et al. (2009) and Uno et al. (2012)). The contribution of such constitutive activity towards the intrinsic clearance of chemicals has served as a conservative measure of the chemical's bioaccumulation potential (OECD 2018a). Therefore, we evaluated enzyme kinetics at basal levels – that is by using fish maintained under controlled environmental conditions for the isolation of S9 sub-cellular fractions and by using cell lines without chemical exposure. Both S9 fractions and cell lines were directly evaluated for the constitutive presence and activity of the different phase I and II biotransformation enzymes by employing the bioassays described in Section Bioassays.

#### Induced activity in cell lines

Experiments using rainbow trout cell lines also explored the inducibility of biotransformation enzymes. The rationale for conducting these evaluations was well-established knowledge of the ability of many chemicals in the environment to induce the expression and subsequent activity of biotransformation enzymes (Schlenk et al. 2024). For this purpose, cells were pre-exposed to known inducers prior to conducting enzyme activity bioassays (Table 1).

**Table 1:** Summary of bioassays established for the evaluation of biotransformation enzyme kinetics in S9 sub-cellular fractions and rainbow trout cell lines

Enzyme	Experimental Model	Condition	Inducer	Enzyme Substrate	Substrate Concentration
Cyp1a	BT-S9	Basal	–	7-ethoxy resorufin (7-ER)	0.05—100 µM
	RT-S9	Basal	–		
	RT cell lines	Basal & Induced	β-naphthoflavone		0.1—25 µM
Cyp2b	BT-S9	Basal	–	7-pentoxyresorufin (7-PR)	0.05—100 µM
	RT-S9	Basal	–		
	RT cell lines	Basal & Induced	β-naphthoflavone		0.5—50 µM
Cyp3a	BT-S9	Basal	–	7-benzyloxy-4-trifluoromethylcoumarin (BFC)	0.05—100 µM
	RT-S9	Basal	–		
	RT cell lines	Basal & Induced	Benzo[a]pyrene	dibenzylfluorescein (DBF)	0.5—25 µM
Gst	BT-S9	Basal	–	1-chloro-2,3-dinitrobenzene (CDNB)	0.05—100 mM
	RT-S9	Basal	–		
	RT cell lines	Basal	–		0.5—100 µM
Ugt	BT-S9	Basal	–	*p*-nitrophenol	0.1—200 µM
	RT-S9	Basal	–		
	RT cell lines	Basal	–		

Specifically, the aryl hydrocarbon receptor (Ahr) agonist β-naphthoflavone (βNF) was used to induce Cyp1a activity (Franco et al. 2018; Heinrich et al. 2014). RTL-W1, RTgutGC and RTbrain cells received a 0.1 μM concentration of βNF, whereas RTgill-W1 cells received a 0.01 μM βNF concentration. These concentrations were selected from preliminary assessments of cytotoxicity and induction assays (Fig. S1 and S2), and from the reports by Franco et al. (2018). βNF also induced Cyp2b-like activity; therefore, subsequent assays employed βNF using the same concentrations outlined before. Similarly, based on reports by Stresser et al. (2000) and Creusot et al. (2015), the polycyclic aromatic hydrocarbon benzo[a]pyrene (BaP) was used to induce the activity of Cyp3a. BaP has been shown to induce Cyp3a-like activity through the pregnane X receptor (*Pxr*) (Luckert et al. 2013). All cell lines received a 0.1 μM BaP concentration as this compound showed lower toxicity compared to βNF in rainbow trout cell lines (Franco et al. 2018; Stadnicka-Michalak et al. 2018). Both βNF and BaP stock solutions were prepared in DMSO, and the dosing solutions were subsequently prepared in L-15 medium as to not exceed 0.5% (v/v) DMSO. In all cases, cells were returned to the incubator for a 24 h exposure period prior to measuring enzymatic activity.

Although experimental trials with BaP were also conducted to evaluate the inducibility of Gst based on the reports by Santana et al. (2018), no significant increases in activity were observed. Furthermore, due to the lack of detectable constitutive Ugt activity in the RT cell lines and taking into account the observations from Christen and Fent (2014) in a zebrafish cell line, no further induction experiments were performed for phase II biotransformation enzymes.

#### Bioassays

Phase I enzymatic reactions using S9 sub-cellular fractions were established based on the methods reported by Smith and Wilson (2010) and Franco et al. (2024). For all Cyp isoforms, reactions consisted in 1 mg/mL S9 protein, 2 mM NADPH, and 100 mM KPO_4_ buffer (pH 7.8), and they were monitored for 30 min. The ethoxyresorufin-O-deethlyase, (EROD), pentoxyresorudin-O-deethylase (PROD), and benzyloxy-4- trifluoromethylcoumarin-O-debenzyloxylase (BFCOD) assays were used for measurements of Cyp1a, Cyp2b-, and Cyp3a-like activities, respectively. The specific enzyme substrates and the concentration ranges used for each bioassay are shown in Table 1.

Cyp activities in cell lines were also measured by similar approaches as when using S9 fractions but adapted for cell monolayers and without the addition of enzyme co-factors (Clemons et al. 1996). Cell monolayers were washed with 100 μL PBS prior to the addition of the respective enzyme substrates. As with S9 fractions, the EROD and PROD assays were performed for measurements of Cyp1a and Cyp2b-like activities, respectively. However, while the BFCOD assay indicated significant Cyp3a-like activity, this did not follow Michaelis–Menten kinetics, as the product signal continued to increase even at significantly higher BFC concentrations (Fig. S3). Therefore, Cyp3a-like activity was alternatively assessed via the conversion of dibenzylfluorescein (DBF) to fluorescein (Donato et al. 2004; Stresser et al. 2000). It is important to point out that while this method monitors a reaction primarily catalyzed by Cyp3a isoforms, Cyp2c enzymes may also contribute (Salminen et al. 2011; Stresser et al. 2000). The substrate concentration ranges used in each bioassay are outlined in Table 1.

Constitutive phase II biotransformation kinetics were also evaluated in S9 sub-cellular fractions and cell lines. In S9 fractions, Gst activity was evaluated colorimetrically at 340 nm following the method by Habig et al. (1974), using 1-chloro-2,4-dinitrobenzene (CDNB) as substrate, and adapted for 96-well plates. Reactions consisted of 0.1 mg/mL S9 protein, 5 mM reduced glutathione (GSH), and 100 mM KPO_4_ buffer. In cell lines, Gst activity was also characterized through the conjugation of CDNB adapted for cell monolayers – that is without the addition of GSH (Tierbach et al. 2020). In both systems, the resulting Gst activity was calculated with a conjugated CDNB molar extinction coefficient of 9.6 mM^−1^, adapted for 96-well plates (5.03 mM^−1^).

The activity of Ugt in S9 fractions was determined from the glucuronidation of *p*-nitrophenol, following the protocol by Ladd et al. (2016) adjusted for 96-well plates. Reactions consisted of 1 mg/mL S9 protein, 25 µg/mL alamethicin, 2 mM uridine 5’-diphosphoglucuronic acid trisodium salt (UDPGA), and 100 mM KPO_4_ buffer. In cell lines, the addition of co-factors was not necessary. In both systems, reductions in absorbance were measured at 400 nm.

Lastly, the kinetics of all detected enzymes in the cell lines was normalized by the protein content in each well using the method described by Lorenzen and Kennedy (1993). After kinetic measurements, cells were washed with PBS and lysed with a 0.1% TritonX solution. A 0.3 mg/mL fluorescamine solution in acetonitrile was then added to the resulting lysate, and the protein content was quantified by constructing a standard curve with BSA. Further details about all enzyme activity bioassays in S9 fractions and cell lines are provided in the SI (*Sect. 1.2*).

#### Data analyses

Non-linear regression analyses were employed for all datasets after plotting the enzymatic activity as a function of the substrate concentration. Substrate concentrations that led to significantly lower enzymatic activity after the maximum reaction rate occurred were removed from the analysis if they fell outside of a 95% confidence interval of the fitted regression line and deviated more than 10% from the point with the highest reaction rate. Once points were removed, Michaelis–Menten kinetic parameters, including maximum reaction rates (*V*max) and rate constants (*Km*), were estimated using GraphPad Prism (version 9.3.1) for Windows.

## Results

### Organ-specific enzyme kinetics across in vitro systems

#### Phase I biotransformation

**Fig. 1:**
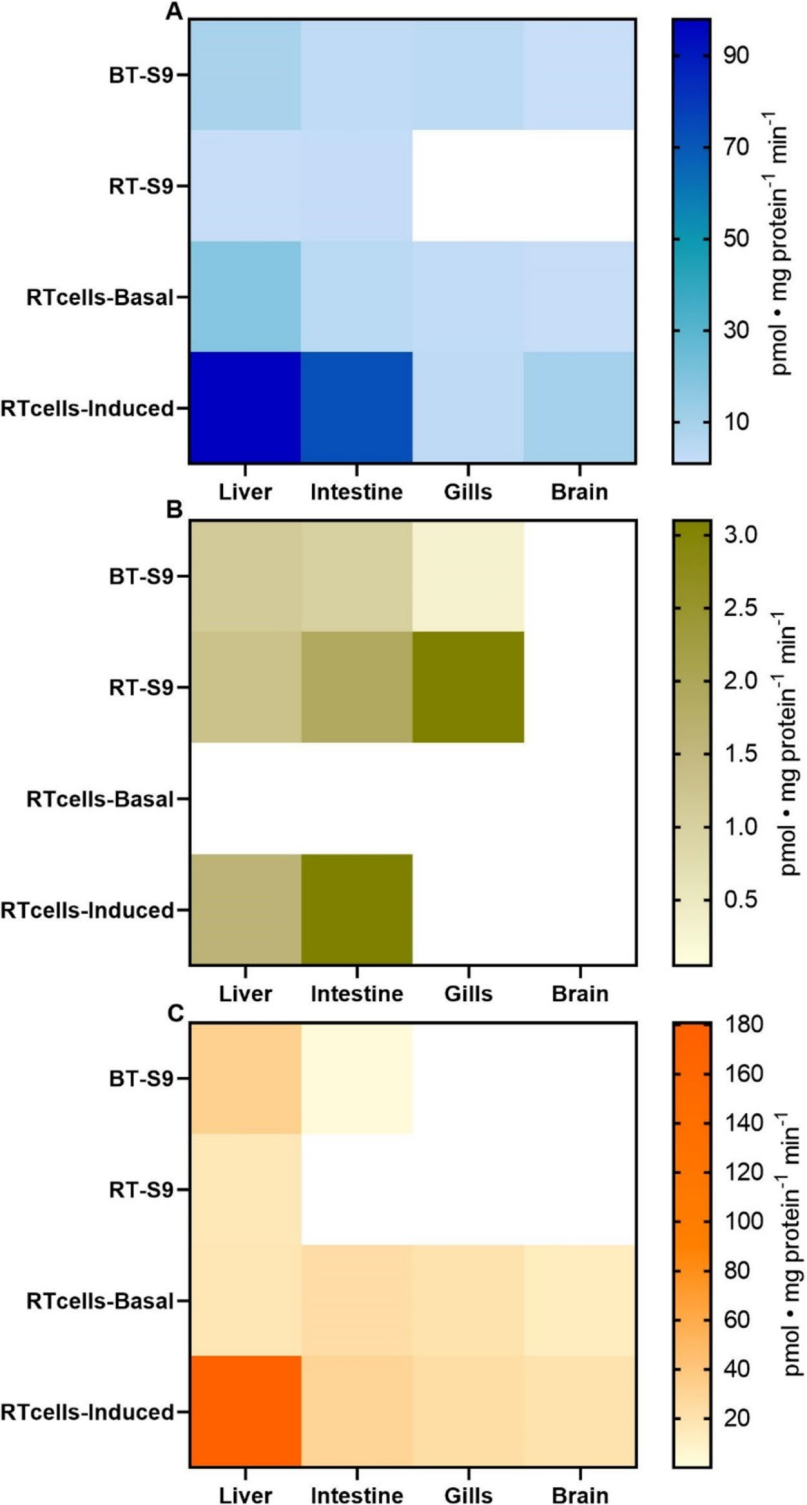
Maximum reaction rates (*Vmax*) derived from Michaelis–Menten kinetics of A) Cyp1a, B) Cyp2b-, and C) Cyp3a-like activities in S9 sub-cellular fractions and cell lines isolated from the liver, intestine, gills and brain of brown trout (*Salmo trutta*) and rainbow trout (*Oncorhynchus mykiss*). *Vmax* values were obtained from plotting mean enzymatic activity of three S9 pools with two technical replicates each, whereas for cell lines *Vmax* values were derived from a minimum of two biological replicates with six technical replicates each (Fig. S4 and S5). Empty (white) cells indicate that no activity was detected or that *Vmax* could not be estimated given that the observed activity did not follow Michaelis–Menten kinetics (in the case of Cyp1a in RT-S9 from gills and brain)

Constitutive phase I biotransformation activity was observed among different organs and the two species, even though enzymatic affinities towards standard substrates (*Km*) and maximum reaction rates (*Vmax*) presented significant differences (Table 2). Among the different organs, Cyp1a displayed its highest *Vmax* in the liver across all test systems (Fig. 1A). Contrarily, the kinetics in the other organs indicated significant reductions in 7-ER affinities and significantly lower *Vmax* when compared to the liver. In general, Cyp1a activity across extrahepatic organs was the highest in the intestine, followed by the brain, and the gills. However, in RT-S9, Cyp1a only displayed Michaelis–Menten kinetics in the liver and intestine (Fig. 1A; Fig. S4 B). When directly comparing the two salmonid species, BT-S9 displayed a significantly higher Cyp1a than RT-S9. In terms of inducibility, the highest levels were observed in the RTL-W1 liver cell line, followed by RTgutGC, RTbrain, and RTgill-W1, in that order (Fig. 1A, bottom row; Fig. S5 B).

**Table 2:** Estimated Michaelis–Menten parameters and goodness of fit (R^2^) of predicted kinetics of phase I and II biotransformation enzymes in S9 sub-cellular fractions isolated from the liver, intestine, gills, and brain of brown trout (*Salmo trutta*) and rainbow trout (*Oncorhynchus mykiss*), and in the cell lines RTL-W1, RTgutGC, RTgill-W1 and RTbrain. Rate constants (*Km*) are given in µM (mM for Gst in BT-S9 and RT-S9) and maximum reaction rates (*Vmax*) are given in pmol mg protein^−1^ min^−1^ (nmol mg protein^−1^ min^−1^ for Gst in BT-S9 and RT-S9). Gst: Glutathione S-transferase. Ugt: UDP-glucuronosyl transferase

	**Liver**											
	**BT-S9**			**RT-S9**			**RTL-W1**			**RTL-W1 Induced**		
	***Km***	***V*max**	**R**^**2**^	***Km***	***V*max**	**R**^**2**^	***Km***	***V*max**	**R**^**2**^	***Km***	***V*max**	**R**^**2**^
Cyp1a	1.6	8.9	0.94	0.8	1.0	0.61	0.1	17.7	0.41	0.8	97.9	0.82
Cyp2b	5.7	1.1	0.97	4.9	1.3	0.95	–	–	–	20.7	1.6	0.76
Cyp3a	24.1	32.6	0.97	26.0	16.2	0.64	8.5	17.6	0.67	15.2	180.7	0.78
Gst	0.8	854.1	0.83	0.7	590.7	0.90	18.8	2.1	0.71	–	–	–
Ugt	37.6	393.8	0.89	3.7	416.2	0.48	–	–	–	–	–	–
	**Intestine**											
	**BT-S9**			**RT-S9**			**RTgutGC**			**RTgutGC Induced**		
	***Km***	***V*max**	**R**^**2**^	***Km***	***V*max**	**R**^**2**^	***Km***	***V*max**	**R**^**2**^	***Km***	***V*max**	**R**^**2**^
Cyp1a	76.7	3.0	0.94	7.2	1.7	0.60	0.3	4.4	0.67	0.9	72.3	0.70
Cyp2b	3.9	1.0	0.96	25.0	1.9	0.81	–	–	–	20.7	3.1	0.77
Cyp3a	29.9	2.3	0.89	–	–	–	7.0	23.8	0.87	12.8	30.0	0.72
Gst	0.7	328.2	0.82	0.6	304.6	0.90	2.0	0.9	0.22	–	–	–
Ugt	0.74	193.2	0.10	10.9	864.0	0.57	–	–	–	–	–	–
	**Gills**											
	**BT-S9**			**RT-S9**			**RTgill-W1**			**RTgill-W1 Induced**		
	***Km***	***V*max**	**R**^**2**^	***Km***	***V*max**	**R**^**2**^	***Km***	***V*max**	**R**^**2**^	***Km***	***V*max**	**R**^**2**^
Cyp1a	33.9	3.6	0.92	–	–	–	0.1	2.4	0.48	0.2	3.4	0.60
Cyp2b	0.6	0.3	0.94	3.2	3.1	0.92	–	–	–	–	–	–
Cyp3a	–	–	–	–	–	–	5.2	19.5	0.79	6.5	23.5	0.64
Gst	0.6	554.1	0.84	0.4	429.5	0.89	7.7	0.4	0.58	–	–	–
Ugt	–	–	–	–	–	–	–	–	–	–	–	–
	**Brain**											
	**BT-S9**			**RT-S9**			**RTbrain**			**RTbrain Induced**		
	***Km***	***V*max**	**R**^**2**^	***Km***	***V*max**	**R**^**2**^	***Km***	***V*max**	**R**^**2**^	***Km***	***V*max**	**R**^**2**^
Cyp1a	49.7	0.9	0.89	–	–	–	0.7	1.0	0.43	0.8	9.7	0.84
Cyp2b	–	–	–	–	–	–	–	–	–	–	–	–
Cyp3a	–	–	–	–	–	–	8.0	13.0	0.86	20.9	19.9	0.88
Gst	0.7	330.6	0.92	0.9	209.5	0.92	4.8	1.2	0.73	–	–	–
Ugt	–	–	–	–	–	–	–	–	–	–	–	–

Cyp2b-like activity followed Michaelis–Menten kinetics in both BT-S9 and RT-S9 isolated from the liver, intestine and gills (Fig. S4 C/D). No Cyp2b-like activity was observed in brain S9 fractions in neither species. Cyp2b in S9 fractions from the liver and intestine displayed similar *Vmax*. However, *Vmax* showed important differences among species. Particularly for the RT-S9 from the gills, as *Vmax* was approximately 10-times higher than those observed in BT-S9 from the gills (Fig. 1B; Fig. S4 D). In addition, the highest affinity for the 7-PR substrate was observed in gill S9 fractions of both species (Table 2). Cyp2b-like activity in the cell lines was only detected in RTL-W1 and RTgutGC upon induction (Fig. 1B, bottom row; Fig. S5 E). RTgutGC displayed approximately twice the *Vmax* of RTL-W1.

Lastly, Cyp3a-like activity was detected in BT-S9 from the liver and intestine, but only in RT-S9 from the liver (Fig. 1C; Fig. S4 E/F). Both BT-S9 and RT-S9 from the liver showed similar affinity towards BFC (Table 2). However, the overall *Vmax* in the hepatic RT-S9 was approximately half of that of BT-S9 (Fig. 1C; Fig. S4 E/F). Lastly, while BT-S9 from the intestine displayed a slight decrease in affinity, its *Vmax* was approximately 14-times lower than S9 from the liver. Despite the observations for BT-S9 and RT-S9, Cyp3a-like activity was displayed in all of the four cell lines tested, both under constitutive and induced conditions (Fig. 1C; Fig. S5 C/D). Here, Cyp3a inducibility also followed particular patterns, as while the highest constitutive *Vmax* occurred in RTgutGC cells, RTL-W1 cells showed the highest *Vmax* upon induction (Fig. 1C; Fig S5 E). Altogether, Cyp3a in both systems was the most active in the liver followed by the intestine, the gills, and the brain in that order.

#### Phase II biotransformation

**Fig. 2:**
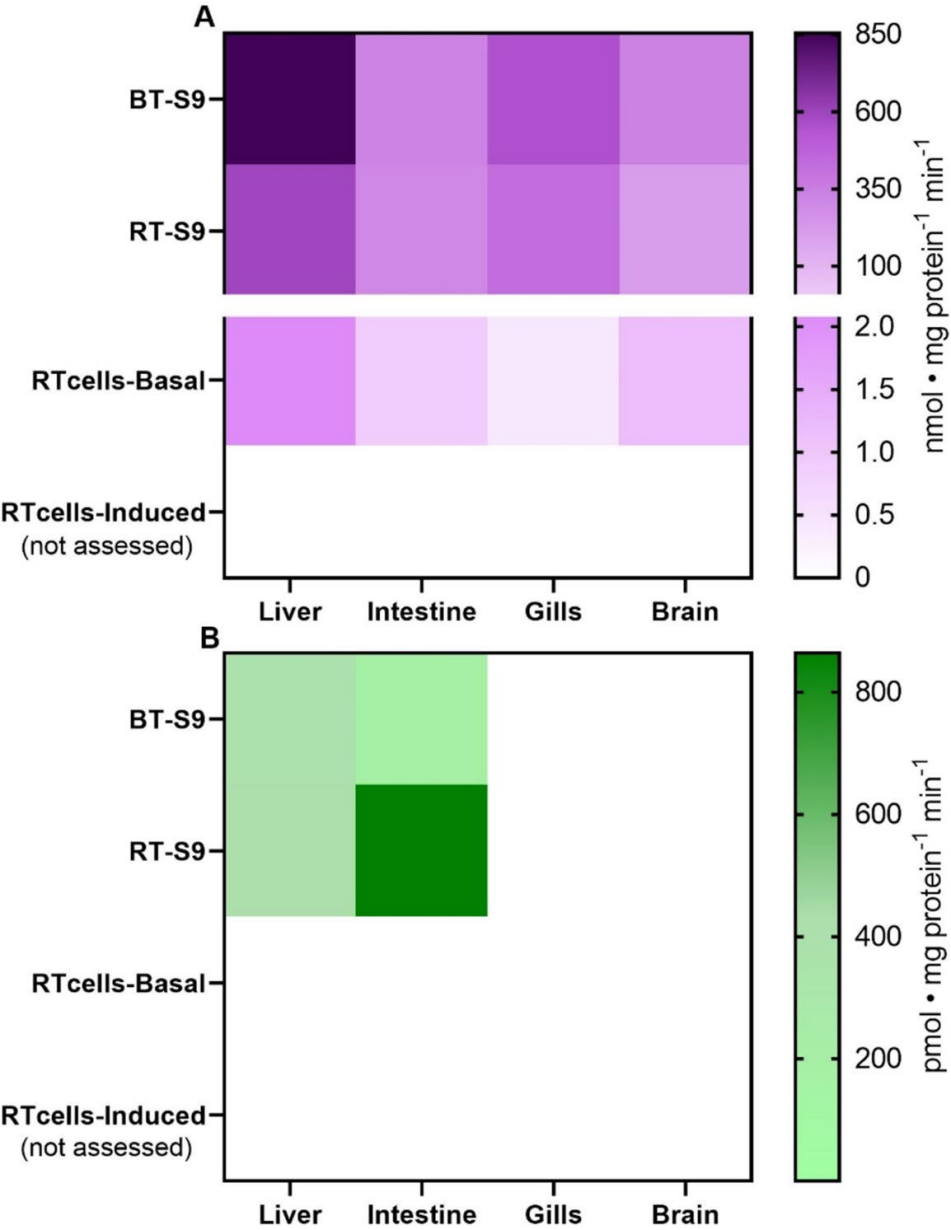
Maximum reaction rates (*Vmax*) derived from Michaelis–Menten kinetics of A) glutathione-S-transferase (note the scale break for easier visualization of the magnitude differences between S9 fractions and cell lines) and B) UDP-glucuronosyltransferase activities in S9 sub-cellular fractions and cell lines isolated from the liver, intestine, gills and brain of brown trout (*Salmo trutta*) and rainbow trout (*Oncorhynchus mykiss*). *Vmax* values were obtained from plotting mean enzymatic activity of three S9 pools with two technical replicates each, whereas for cell lines *Vmax* values were derived from a minimum of two biological replicates with six technical replicates each (Fig. S6 and S7). Empty (white) cells indicate that no activity was detected or that activity was not assessed (in the case of induction of phase II enzymes in RT cell lines)

Phase II enzymes also displayed constitutive, organ-specific activity in both S9 fractions and cell lines (Fig. 2A). Michaelis–Menten kinetics for Gst was observed in both BT-S9 and RT-S9 from all organs (Fig. S6 A/B). In BT-S9, the affinity of Gst towards its substrate CDNB remained similar across the different organs tested (Table 2). However, for RT-S9, the gills showed the highest affinity towards CDNB. In terms of *Vmax*, both BT-S9 and RT-S9 showed the same patterns. In decreasing order, Gst activity in both BT-S9 and RT-S9 was the highest in the liver > gills > brain > intestine. BT-S9 from the intestine and brain displayed almost identical values. In cell lines, a similar pattern for Gst activity was observed, with RTL-W1 cells showing the highest *Vmax*, both RTgutGC and RTbrain having similar activities, and RTgill-W1 displaying the lowest *Vmax* (Fig. S7).

Lastly, constitutive Ugt activity was only detected in BT-S9 and RT-S9 from the liver and intestine (Fig. 2B; Fig. S6 C/D). No activity was detected in neither of the cell lines tested. Ugt in the BT-S9 from the liver displayed affinity towards *p*-nitrophenol approximately 50-times lower than the intestinal S9, yet *Vmax* was significantly higher in the liver than in the intestine (Table 2). Contrarily, RT-S9 from the liver presented higher affinity for CDNB than the intestinal S9, but *Vmax* in the liver S9 was approximately half of that measured in intestinal S9.

### RT-S9 vs RT cell lines

In addition to the organ-specific trends for biotransformation enzyme activity, significant differences were observed when directly comparing the performance of RT-S9 with that of the different rainbow trout cell lines. In all cases, Cyp1a displayed both higher affinity and *Vmax* in cell lines than in RT-S9 (Fig. 1A; Table 2). On average, constitutive Cyp1a activity in cells was approximately between 2 – 17 times higher than in RT-S9. This difference was exacerbated upon induction, where Cyp1a experienced a slight decrease in affinity towards the substrate but *Vmax* significantly increased between 43 – 98 times higher than in RT-S9, particularly in RTL-W1 and RTgutGC cells.

A comparison of constitutive Cyp2b-like activity between RT-S9 and cell lines was not possible, given that no basal activity was detected in the cells. However, upon induction, liver and intestinal cell lines showed similar *Vmax* than those in RT-S9 isolated from the same organs (Table 2). For Cyp3a-like activity, a RT-S9 vs cell line comparison was only possible for the liver. Constitutive Cyp3a-like activity was very similar between RT-S9 and RTL-W1 (16.2 and 17.6 pmol • mg protein^−1^ min^−1^, respectively), yet RTL-W1 cells displayed a *Vmax* approximately 11-times higher than the basal Cyp3a-like activity in RT-S9 upon induction.

In contrast to phase I biotransformation, where activities were higher in the cell lines, constitutive Gst activities in RT-S9 were significantly higher than in cells. *Vmax* in RT-S9 were between 175- and 1000-times higher than those observed in the different cell lines. Similarly, the affinity of Gst towards CDNB was higher (between 3 and 27 times) in RT-S9 than in cell lines across all organs.

## Discussion

### Phase I biotransformation drives the biotransformation capacity of fish *in vitro* systems

The present study reports important evidence for the biotransformation capacity of fish in vitro systems, namely S9 sub-cellular fractions and permanent cell lines, when assessing organ-specific kinetics of phase I and II biotransformation enzymes. Our observations for the significant Cyp biotransformation activity across organs and test systems corroborate the value of this superfamily of enzymes for the biotransformation capacity of fish (Goksøyr 1995; Sarasquete and Segner 2000; Schlenk et al. 2024). The need to process endogenous and exogenous compounds that fish may encounter throughout their life cycle is likely responsible for the evolution of diverse biotransformation pathways. Indeed, more than 100 Cyp isoforms have been previously described in fish (Uno et al. 2012), and such diversity could be fundamental for maintaining the functionality of biotransformation processes, particularly in organisms inhabiting polluted environments (Franco et al. 2025).

In the context of phase I biotransformation, the presence and activity of different Cyp enzymes may be related to potential compensatory mechanisms allowing different Cyp isoforms to support specific reactions involving different xenobiotics. For example, Ankley et al. (2007) reported significant increases in the expression of Cyp11 and Cyp17 in fathead minnows upon exposure to ketoconazole, a fungicide known to inhibit a variety of other Cyps in vertebrates. In another study, Franco et al. (2022a) reported increases in Cyp2b-like activity in Gulf killifish upon BaP exposure when Cyp1a was downregulated. In fish cell lines, a similar scenario was discussed by Stadnicka-Michalak et al. (2018), who reported BaP biotransformation in the rainbow trout gill cell line RTgill-W1, despite its rather low Cyp1a activity. We moreover demonstrated that every cell line tested is equipped, not only with Cyp1a, but with an active Cyp3a-like pathway as well, an important route for the biotransformation of both legacy contaminants and micropollutants (e.g. pharmaceuticals) (Schlenk et al. 2024). This pathway may be fundamental when other major biotransformation pathways display low or no activity, as observed for Cyp1a and Cyp2b in RTgill-W1 cells. Indeed, potential overlapping specificities among different biotransformation enzymes could aid in maintaining the biotransformation capacity and plasticity of organisms (Tierbach et al. 2020).

When looking at specific biotransformation pathways across organs, our results further indicated that, while Cyp1a was most prominent in the liver, all extrahepatic organs displayed significant activity both in S9 fractions and cell lines. This is important because our observations as well as those from others showed that Cyp1a-mediated biotransformation in extrahepatic organs is active and plays a major role in dealing with chemical exposure in vivo (Burkina et al. 2021; Santos and dos Reis Martinez 2020), and when evaluated in sub-cellular fractions (Franco et al. 2024) and cell cultures (Behrens et al. 2001; Stadnicka-Michalak et al. 2018). Unfortunately, direct comparisons of enzyme kinetics and magnitude of activity between our results and those from the referenced literature are not entirely possible due to differences in how bioassays were conducted (e.g., use of different substrates and substrate concentrations).

However, the biotransformation of chemical pollutants in fish does not depend on the presence and activity of the Cyp1a family alone. In this context, our observations regarding the detection of Cyp2b- and Cyp3a-like activities at basal and induced levels across organs are important in further characterizing the biotransformation capabilities of fish. The regulation of Cyp2b enzymes directly depends on the functionality of the constitutive androstane receptor (*Car*) (Chirulli et al. 2005; Iwata et al. 2002), whereas Cyp3a enzymes depend on the activity of the *Pxr* (Luckert et al. 2013; Luo et al. 2004). Both pathways are important in the processing of chemical pollutants that act as agonists of these Cyp enzymes.

In comparison with cell lines, however, the observations suggesting limited functionality and a potential absence of *Car* and *Pxr* pathways in BT-S9 and RT-S9 from the different organs do not necessarily indicate that S9 sub-cellular fractions have limited applicability when assessing Cyp2b or Cyp3a agonists. This specific observation in our study may correspond to a lower expression and activity of these pathways in the specific individuals employed in S9 fraction isolation, as was discussed by Franco et al. (2024), and given that previous reports have indicated e.g. significant Cyp3a-like activity in rainbow trout liver and intestine at levels higher than the ones observed in our study (Saunders et al. 2020). In this context, our results highlight that cell lines, in particular, are apparently able to display functional *Car*-like and *Pxr*-like pathways across different organs and could support biotransformation assessments for different classes of chemicals without animal use.

### Phase II biotransformation is more prominent in S9 fractions than in cell lines

The assessment of phase II biotransformation enzymes elucidated organ-specific patterns in both test systems. Constitutive Gst activity has been reported in different fish species (Dominey et al. 1991; Hayes and Pulford 1995; Tierbach et al. 2018), and its presence is suggested to arise from its involvement in multiple biological processes beyond biotransformation, such as redox reactions and antioxidant pathways (Lenartova et al. 2008). In our study, Gst activity in S9 fractions and cell lines displayed Michaelis–Menten kinetics in all organs, yet the magnitude of this activity differed significantly among the test systems. Both BT-S9 and RT-S9 from all organs displayed higher Gst activity than their counterpart cell lines. Tierbach et al. (2020) provided a similar observation suggesting that Gst expression in a zebrafish cell line (PAC2) differed in expression patterns as compared to embryos and adult zebrafish, despite characterizing a fully functional Gst pathway in the cells. Our observations related to the differential magnitude of Gst activity in S9 fractions and cell lines are then likely the result of a potential reduction of metabolic potential in cell lines when compared to freshly isolated tissues (Bols et al. 2005; Thibaut et al. 2009). However, the fact that Gst could not be induced to levels beyond the constitutive activity speaks for maximal expression levels also in the cell lines, as was also shown in other in vitro and in vivo studies with zebrafish (Tierbach et al. 2020, 2018).

Glucuronidation also represents a major phase II biotransformation pathway in fish (see review by Clarke et al. (1991)), and although most of the Ugt-associated pathways have been thoroughly characterized for zebrafish (Christen and Fent 2014; Wang et al. 2014), previous studies have indicated significant hepatic Ugt activity in salmonids, catfish, and mummichogs (Gaworecki et al. 2004; Hooft et al. 2021; Ladd et al. 2016; Mortensen and Arukwe 2007). In our study, Ugt activity followed Michaelis–Menten kinetics in both BT-S9 and RT-S9 from the liver and intestine, similar to the observations from Saunders et al. (2020). These results highlight the important role of Ugt in supporting glucuronidation reactions both hepatically and extrahepatically. However, Ugt activity was not detected in neither of the RT cell lines. Christen and Fent (2014) reported similar observations for zebrafish, where Ugt expression was not detected in the ZFL zebrafish cell line even after induction with BaP, despite displaying high Ugt expression in embryos. More recently, Ankley et al. (2024) observed that glucuronidation activity in the RTL-W1 cell line was low when assessing the biotransformation of tire chemicals. In our study, the lack of Ugt activity may be also related to a significantly low expression of Ugt-associated genes in the rainbow trout cells, and a potential divergence in Ugt potency compared to freshly isolated tissue. Undetectable Ugt activity may have also resulted from potential differences in membrane transport when evaluating particular substrates, considering the observations by Thibaut et al. (2009) who reported glucuronidation activity in RTL-W1 cells upon exposure to 1-naphthol and testosterone. In such case, the absence in activity could be explained by the inability of the substrate *p*-nitrophenol to successfully reach the cytosol and interact with Ugt isoforms within the established incubation period, as was discussed by Fay et al. (2016) when evaluating hepatocytes in biotransformation studies. Therefore, further induction experiments employing different chemical inducers may be necessary to explore Ugt activity in fish cell lines.

### Important considerations when selecting S9 fractions or cell lines for biotransformation studies

When directly comparing the in vitro test systems employed in our study, an important consideration is the presence of intact cells in cell lines vs*.* a “cocktail” of enzymes for S9 fractions. When employing S9 fractions, it is assumed that substrates are readily available for enzymes as membranes are disrupted during homogenization and lysis procedures. Contrarily, membrane transport represents a critical aspect when assessing the applicability of cell cultures in biotransformation studies as the need to first pass the cell membrane may modify biotransformation rates whereas, without such a barrier, as in S9 fractions, selected substrates may be rapidly transformed (Fay et al. 2016). In the same vein, enzyme–substrate interactions could be modulated due to the activity of e.g. ATP-binding cassette (ABC) transporters as they have been shown to significantly influence membrane transport in cell lines, thus altering dynamics of uptake and retention of chemicals and their biotransformation products (Fischer et al. 2011). In addition, the adsorption of substrates to the lipid bi-layer of the cell membranes could reduce their availability to enzymes found in the intracellular compartment, as discussed in previous studies upon conducting bioaccumulation experiments with ionizable chemicals and fish cell lines (Balk et al. 2023, 2024). This is also why, in comparison to S9 fractions, cell lines require longer incubation times to allow for enough substrate to cross the cell membranes and become available for the biotransformation enzymes (Stadnicka-Michalak et al. 2018). By the same token, cell lines do allow for much longer incubation times (days vs. h for S9), which could be particularly advantageous for studying chemicals that are slowly transformed.

Another reason for the divergence observed between test systems could be the need to add enzymatic co-factors when employing S9 fractions, which, to some extent, may interfere with enzyme-chemical interactions if, for example, S9 fractions from different organs and species require different co-factor concentrations (Foster et al. 2011; OECD 2018a). Cell cultures, on the other hand, are self-dependent when it comes to their ability to produce the necessary co-factors and support enzymatic reactions. Thus, the need to optimize co-factor levels is not required for cell-based bioassays and the cost for the assays themselves is thereby reduced.

Lastly, when assessing the suitability of in vitro systems for biotransformation studies, it is imperative to consider the inducibility of biotransformation pathways, which was clearly demonstrated in the present study when assessing enzyme activity in rainbow trout cell lines. Biotic, chemical, and environmental factors are known to influence the degree of biotransformation activity in an organism (Cerveny et al. 2021; Schlenk et al. 2008; Uno et al. 2012). The observations from our study as well as from previous assessments (Creusot et al. 2015; Heinrich et al. 2014; Santos et al. 2018; Stadnicka-Michalak et al. 2018) provide significant evidence of the differential pre- and post-induction activity of biotransformation enzymes. Induction experiments with different chemicals and the description of the molecular mechanisms driving specific induction patterns, while falling outside of the scope of the present study, represent an important avenue of research in support of environmental risk assessments. It must be kept in mind, however, that while it is possible to explore the inducibility of biotransformation pathways using S9 fractions, donor animals must be previously exposed to inducers (Burkina et al. 2012; Ortiz-Delgado et al. 2008). Such additional animal experimentation increases the costs and logistics of biotransformation studies as well as the need for animals when the aim is to reduce animal use. Contrarily, cell-based bioassays allow for the induction of specific biotransformation enzymes by pre- (or also co-) exposing cells to known chemical inducers, as shown in our study, prior to the evaluation of biotransformation activity without significant additional resources. This advantage highlights cell lines as suitable tools to explore the inducibility of biotransformation pathways and to increase the environmental relevance of cell-based bioassays. Adding to these considerations is the fact that cell lines, once established, express biotransformation enzyme activity consistently, even when cultured for decades, as can be illustrated with the RTL-W1 cell line, which was first established by Lee et al. (1993). Enzymatic activity in S9 fractions, on the other hand, is likely to vary with e.g. donor animals, sex, life cycle, and season (Johanning et al. 2012).

## Conclusion

Our study falls in line with previous reports highlighting differential activity of biotransformation enzymes across organs as a critical component of the overall biotransformation capacity of fish (Burkina et al. 2021; Christen and Fent 2014; Franco et al. 2024; Moller et al. 2014; Ortiz-Delgado et al. 2008; Yuan et al. 2013). The observations presented here highlight the need to consider biotransformation processes more comprehensively by taking into account different organs, test systems, and environmental influence, like pre-exposure to chemicals and the subsequent inducibility of biotransformation pathways. The experimental approaches described could be also applied to other fish species to address priority questions related to mechanisms of defense against chemical exposure and key traits driving species sensitivity to pollution. While these biochemical approaches focusing on enzyme kinetics may only provide a snapshot of the overall biotransformation capacity of fish, they could be also integrated with gene and protein expression analyses to help in the identification and mapping of major biotransformation pathways, especially if OMICS-based technologies are also implemented to guide the development of specific bioassays.

The present study also underlines that important experimental considerations must be taken when selecting appropriate in vitro systems for biotransformation studies. However, we demonstrated that both test systems, S9 fractions and cell lines, are able to display biotransformation pathways and allow for the evaluation of organ-specific biotransformation ability under basal and induced conditions. Therefore, exploiting their applicability within biotransformation studies could elucidate how different enzymatic pathways actually contribute to chemical clearance, particularly for chemicals that may display differential affinity for phase I and II biotransformation pathways. Both approaches allow for a mechanistic understanding of biotransformation activity that is unlikely to be gained from traditional in vivo experimentation. As such, comprehensive investigations of biotransformation pathway kinetics in fish must continue in order to support research avenues aimed to understand the impact of chemical pollution in aquatic ecosystems.

## Data availability statement

Data are available upon request.

## Supplementary Information

Below is the link to the electronic supplementary material.

Supplementary file1 (DOCX 4479 KB)
