# Supplementary material for: Comparative characterization of organ-specific phase I and II biotransformation enzyme kinetics in salmonid S9 sub-cellular fractions and cell lines
Source: Cell Biol Toxicol. 2025 Jan 28;41(1):37. doi: 10.1007/s10565-025-09992-8 (PMC11775053; doi:10.1007/s10565-025-09992-8)
Supplement: Supplementary file 1 — Supplementary file1 (DOCX 4479 KB) [file 10565_2025_9992_MOESM1_ESM.docx]

**Comparative characterization of organ-specific phase I and II biotransformation enzyme kinetics in salmonid S9 sub-cellular fractions and cell lines**

Baptiste P. M. Martin^1^, Marco E. Franco^1^, Kristin Schirmer^1,2^

*^1^ Department of Environmental Toxicology, Swiss Federal Institute of Aquatic Science and Technology, Eawag, 8600 Dübendorf, Switzerland*

*^2^ Department of Environmental Systems Science, ETH Zürich, 8092 Zürich, Switzerland*

**Supplemental Information**

1. **Materials and methods 2**
   1. Isolation of S9 sub-cellular fractions 2
   2. Enzyme activity bioassays 3
      1. *S9 sub-cellular fractions* 3
      2. *Cell lines* 4
2. **Tables and Figures**  **8**
3. **References 14**
4. **Materials and Methods**
   1. Isolation of S9 sub-cellular fractions

Liver S9 sub-cellular fractions were prepared following standard procedures (Johanning et al., 2012; OECD, 2018). Upon dissection, the liver was perfused through the hepatic vein with approximately 30 mL of chilled perfusion buffer (Hanks’ balanced solution, 10 mM HEPES, and 2 mM EDTA, pH 7.8), excised, and weighed. Livers from each fish pool were then minced and placed in two volumes of ice-cold homogenization buffer (50 mM Tris-HCL, 150 mM KCl, 2 mM EDTA, 1 mM DTT, and 250 mM sucrose, pH 7.8).

Intestinal S9 fractions were prepared following the protocols described in McElroy and Kleinow (1992) and Saunders et al. (2020). Upon dissection, whole intestines were rinsed with clearing buffer and the anterior intestine was cut longitudinally to expose the intestinal mucosa. A combination of scalpels and glass microscope slides were used to scrape the mucosa into 15 mL conical tubes containing 10 mL of chilled homogenization buffer. The resulting suspensions were then centrifuged (2,000 g for 5 min at 4 °C), and the cell pellet was resuspended in 5 mL of homogenization buffer.

The preparation of S9 fractions from the gills followed the protocol by Gomez et al. (2010), with additional clean-up procedures for blood removal prior to homogenization. The dissected gill arches were placed in petri dishes over ice and with approximately 5 mL of clearing buffer. The tissue was cleared of remaining blood and blood cloths using additional clearing buffer and forceps, and then transferred to a clean petri dish with homogenization buffer. Clean arches were minced into 2-3 mm pieces and pooled into conical tubes with two volumes of chilled homogenization buffer.

Brain S9 sub-cellular fractions were isolated from whole brains. The organs were exposed after a longitudinal cut on the cranium. Brains were carefully removed from the cranial cavity with forceps and pooled into 15 mL conical tubes containing two volumes of homogenization buffer.

Upon transport to the laboratory, the organ suspensions were homogenized with a digital ULTRA-TURRAX® homogenizer (IKA^®^-Werke GmbH & Co. KG, Staufen, DE). The resulting homogenates were centrifuged 13,000 g for 25 min and at 4 °C (UniCn MR, Herolab GmbH, Wiesloch, DE), and the supernatants were then collected and transferred to cryogenic vials in aliquots of 0.25 or 0.5 mL, depending on the resulting S9 volume and to minimize repetitive thawing. Lastly, a small aliquot was used to determine total protein content with the Coomassie blue assay (Bradford, 1976), using bovine serum albumin (BSA) as a standard. All S9 fraction aliquots were stored at -80 °C until assays were performed.

- 1. Enzyme activity bioassays
     1. *S9 sub-cellular fractions*

Cyp1a activity kinetics was measured via the ethoxyresorufin-*O*-deethylase (EROD) bioassay. The reactions consisted in 1 mg/mL S9 protein, 2 mM NADPH, 100 mM KPO_4_ buffer (pH 7.8), and 7-ethoxyresorufin (7-ER) as substrate in methanol (0.5% v/v). The formation of resorufin was monitored for 30 minutes at 25 ℃ and at emission/excitation wavelengths of 535/590 nm. Similarly, Cyp2b-like activity was evaluated via the pentoxyresorufin-*O*-deethylase (PROD) assay measuring the dealkylation of 7-pentoxyresorufin (7-PR; in methanol (0.5% v/v)) to resorufin in reactions similar to those established for Cyp1a. Cyp3a-like activity kinetics were measured via the dealkylation of 7-benzyloxy-4-trifluoromethylcoumarin (BFC; in methanol (0.5% v/v)), respectively. The formation of 7-Hydroxy-4-(trifluoromethyl)coumarin was monitored for 30 min using 1 mg/mL S9 protein and 2 mM NADPH. Emission/excitation wavelengths were 385/500 nm.

Gst activity kinetics was evaluated following the method by Habig et al. (1974) in 96-well plates. Reactions consisted of 0.1 mg/mL, 5 mM reduced glutathione (GSH), 100 mM KPO_4_ buffer, and 1-Chloro-2,3-dinitrobenzene (CDNB) as substrate in ethanol (0.5% v/v). The conjugation of CDNB was monitored for 5 min by colorimetric (absorbance) measurements at 340 nm.

Finally, Ugt activity was monitored for 30 min by measuring absorbance at 400 nm, following the protocol by Ladd et al. (2016) in 96-well plates. Reactions consisted in 1 mg/mL S9 protein, 25 µg/mL alamethicin, 2 mM uridine 5’-diphosphoglucuronic acid trisodium salt (UDPGA), 100 mM KPO_4_ buffer, and *p*-nitrophenol as substrate in acetone (0.5% v/v).

- - 1. *Cell lines*

As with S9 fractions, the activity of phase I and II enzymes was monitored in the four RT cell lines tested but without the addition of enzyme co-factors since intact cells are equipped with the mechanisms to produce their own (Fay et al., 2015). A minimum of two biological replicates with six technical replicates each were used in the analyses of enzyme kinetics with the cell lines. Incubation times for the bioassays were also determined from optimization experiments and ranged between 30 and 120 min.

Cyp1a activity in cell lines was also measured by the EROD assay, using 7-ER as substrate and adapted for cell monolayers (Clemons et al., 1996; Clemons et al., 1994). 7-ER stock solutions were made in methanol, but serial dilutions for working concentrations were then made in PBS as to not exceed > 0.5% methanol (v/v). Briefly, the cell culture medium was carefully removed and the cells were washed with 100 μL PBS prior to the addition of the 7-ER solutions. The plates were immediately transferred to a microplate reader (TECAN infinite M200, Männedorf, CH) and kinetic measurements were taken every 5 min for 30 min at excitation/emission of 535/590 nm.

Measurements of Cyp2b-like activity were conducted via the PROD assay, also using resorufin as product. 7-PR stock solutions were made in methanol and serial dilutions were made in PBS (0.5% methanol v/v). Cells were incubated for 2 h, and measurements were taken at the beginning and at the end of the 2 h period at excitation/emission of 535/590 nm.

Initially, Cyp3a-like activity was evaluated via the benzyloxy-4- trifluoromethylcoumarin-O-debenzyloxylase (BFCOD) assay measuring the dealkylation of 7-benzyloxy-4-trifluoromethylcoumarin (BFC) for 2 h in the RTgutGC cell line. BFC stock solutions were prepared in methanol and serial dilutions were made in PBS (0.5% methanol v/v). RTgutGC cells showed significant Cyp3a-like activity but this did not follow Michaelis-Menten kinetics, given that the product signal continued to display a linear increase even at significantly high concentrations of BFC (500 μM; Fig. S3). Given the observations for RTgutGC cells and the need to apply the same method for direct comparisons between the four cell lines, Cyp3a-like activity were alternatively assessed following the methods by Donato et al. (2004) and Stresser et al. (2000), measuring the conversion of dibenzylfluorescein (DBF) to fluorescein. Previous reports in mammals have suggested that the conversion of DBF to fluorescein is primarily catalyzed by Cyp3a isoforms, yet Cyp2c enzymes may also participate (Salminen et al., 2011; Stresser et al., 2000). Therefore, the method employed was used to measure the biotransformation potential driven by Cyp3a, while potentially considering the contribution of Cyp2c enzymes. Briefly, after a washing step with 100 μL of PBS, cells were incubated with DBF. DBF stock solutions were prepared in methanol and serial dilutions were made in PBS (0.5% methanol v/v). The plates were immediately transferred to the plate reader and the formation of fluorescein was measured kinetically at 5 min intervals for 30 min and at excitation/emission of 485/540 nm.

Gst activity kinetics in cell lines were characterized by measuring the conjugation of CDNB. The bioassay was based on a method reported by Habig et al. (1974) and adapted to cell monolayers. CDNB stock solutions were made in ethanol and subsequently diluted in PBS. After washing with 100 μL of PBS, cells were incubated with CDNB and its conjugation was measured kinetically at 5 min intervals for 30 min at an absorbance wavelength of 340 nm. The quantification of the product was performed with the conjugated CDNB molar extinction coefficient of 9.6 mM^-1^, adapted for 96-well plates (5.03 mM^-1^).

Ugt activity was determined from the glucuronidation of *p*-nitrophenol over time using the method reported by Ladd et al. (2016) for S9 fractions. The method was adapted to cell monolayers in 96-well plates without the addition of 5’-diphosphoglucuronic acid (UDPGA) and alamethicin since co-factor addition was not necessary for the cells and co-exposure of the selected substrate with alamethicin (a pore-forming antibiotic) may cause modifications to the integrity of the plasma membrane and its transport potential (Leitgeb et al., 2007). Cells were incubated with different *p*-nitrophenol concentrations, initially dissolved in acetone for stock solutions, and subsequently diluted in PBS (0.5% acetone v/v). The conjugation of *p*-nitrophenol removes the yellow color that this compound takes in solution. Thus, reductions in absorbance were measured at 400 nm between an initial measurement immediately after adding the substrate and a measurement after 2 h of incubation. The quantification of the conjugated substrate was done using a *p*-nitrophenol standard curve.

Lastly, the protein content of each cell monolayer in each well was determined for normalization purposes. Protein quantification was conducted using the method described by Lorenzen and Kennedy (1993). Briefly, after kinetic measurements of enzyme activity, the substrate solutions were carefully removed and cell monolayers were washed with 100 μL of PBS. Then, 50 μL of a lysis buffer solution (0.1% TritonX in PBS) were added and cells were incubated for 5 min. The resulting cell lysate was diluted by the addition of 100 μL of PBS, followed by 50 μL of a 0.3 mg/mL fluorescamine solution in acetonitrile. The plates were then covered in foil and incubated in a shaker for 5 min prior to measurements of fluorescence at excitation/emission of 360/460 nm. Protein quantification was performed by constructing a standard curve with bovine serum albumin (BSA).

1. **Tables and Figures**

**Table S1.** Average morphometric parameters of brown trout and rainbow trout used in the isolation of S9 sub-cellular fractions. Values correspond to the mean parameter ± SD. K: Fulton’s condition factor. Further information regarding the fish used in the isolation of S9 fractions is available as SI in Franco et al. (2024).

|  |  | **Brown trout** | **Rainbow trout** |
| --- | --- | --- | --- |
| **Length (cm)** |  | 29.6 ± 1.2 | 30.9 ± 1.5 |
| **Weight (g)** |  | 320.1 ± 26.8 | 350.9 ± 42.8 |
| **K** |  | 1.2 ± 0.1 | 1.2 ± 0.1 |
|  |  |  |  |
| **Fractional Organ Weight (g/g whole body)** | **Liver** | 0.01 ± 0.00 | 0.01 ± 0.00 |
|  | **Anterior Intestine** | 0.039 ± 0.007 | 0.053 ± 0.007 |
|  | **Gills** | 0.018 ± 0.003 | 0.022 ± 0.002 |
|  | **Brain** | 0.001 ± 0.000 | 0.001 ± 0.000 |

**Figure S1.** Cytotoxicity of β-naphthoflavone (βNF) on rainbow trout cell lines measured via the CFDA-AM assay for the estimation of membrane integrity. Upon exposing cells in 96-well plates to βNF for 24 h, 200 µL of a 1 µM CFDA-AM solution in PBS were added to each well and plates were incubated for 30 min. Cell viability was then determined via fluorescence measurements at excitation/emission of 493/541 nm. Data are shown as % response relative to non-exposed cells ± SEM, from a minimum of six technical replicates (n = 6 – 12). Dotted, vertical lines correspond to the βNF concentrations used in enzyme induction experiments for RTgill-W1 (0.01 µM) and for the rest of cell lines (0.1 µM).

**Figure S2.** Concentration-dependent activity of ethoxyresorufin-*O*-deethylase (EROD) as proxy for Cyp1a induction in rainbow trout cell lines upon exposure to β-naphthoflavone (βNF). Cells seeded in 96-well plates were dosed with different concentrations of βNF in 200 µL of L-15 culture medium supplemented with 5% FBS per well. Upon a 24 h exposure, EROD activity was measured using a 0.5 µM 7-ethoxyresorufin concentration as described in the materials and methods section. Data are shown as pmol • mg protein^-1^ min^-1^ ± SEM (n = 7 - 12).

**Figure S3.** Dealkylation of 7-benzyloxy-4-trifluoromethylcoumarin (BFC) as proxy for Cyp3a-like activity in RTgutGC cells. Data are shown as mean pmol min^-1^ ± SD of six technical replicates (wells).

**Figure S4.** Michaelis-Menten kinetics of Cyp1a (A, B), Cyp2b (C, D), and Cyp3a (E, F) activities in brown trout and rainbow trout S9 sub-cellular fractions isolated from the liver, intestine, gills and brain. Points on the graphs correspond to mean enzymatic activity ± SEM of three S9 pools with two technical replicates each. Dotted lines indicate a 95% confidence interval. Points shown in red at higher concentrations, indicating a significant reduction of enzyme activity, were not considered in the final estimation of kinetic parameters.

**Figure S5.** Michaelis-Menten kinetics of basal and induced Cyp1a (A, B) and Cyp3a-like (C, D) activities, and induced Cyp2b-like (E) activity in the rainbow trout cell lines RTL-W1, RTgutGC, RTgill-W1, and RTBrain. Points on the graphs correspond to mean enzymatic activity ± SEM of n = 2-3 biological replicates with six technical replicates each. Dotted lines indicate a 95% confidence interval. Points shown in red at higher concentrations, indicating a significant reduction of enzyme activity, were not considered in the final estimation of kinetic parameters.

**Figure S6.** Michaelis-Menten kinetics of glutathione-S-transferase (A, B) and UGP-glucuronosyltransferase (C, D) activities in brown trout and rainbow trout S9 sub-cellular fractions isolated from the liver, intestine, gills and brain. Points on the graphs correspond to mean enzymatic activity ± SEM of three S9 pools with two technical replicates each. Dotted lines indicate a 95% confidence interval. Points shown in red at higher concentrations, indicating a significant reduction of enzyme activity, were not considered in the final estimation of kinetic parameters.

**Figure S7.** Michaelis-Menten kinetics of basal glutathione S-transferase (Gst) activity in the rainbow trout cell lines RTL-W1, RTgutGC, RTgill-W1, and RTBrain. Points on the graphs correspond to mean enzymatic activity ± SEM of n = 2-3 biological replicates with six technical replicates each. Dotted lines indicate a 95% confidence interval. Points shown in red at higher concentrations, indicating a significant reduction of enzyme activity, were not considered in the final estimation of kinetic parameters.

1. **References**

Bradford, M.M., 1976. A rapid and sensitive method for the quantification of microgram quantities of protein utilizing the principle of protein-dye binding. Anal. Biochem. 72, 248-254.

Clemons, J.H., Lee, L.E.J., Myers, M.S., Dixon, D.G., Bols, N.C., 1996. Cytochrome P4501A1 induction by polychlorinated biphenyls (PCBs) in liver cell lines from rat and trout and the derivation of toxic equivalent factors. Canadian Journal of Fisheries and Aquatic Sciences 53, 1177-1185.

Clemons, J.H., van den Heuvel, M.J., Stegeman, J.J., Dixon, D.G., Bols, N.C., 1994. Comparison of toxic equivalent factors for selected dioxin and firran congeners derived using fish and mammalian liver cell lines. Can. J Fish. Aquat. Sci. 51, 1577-1584.

Donato, M.T., Jiménez, N., Castell, J.V., Gómez-Lechón, M.J., 2004. Fluorescence-based assays for screening nine cytochrome P450 (P450) activities in intact cells expressing individual human P450 enzymes. Drug Metab. Dispos. 32, 699-706.

Fay, K.A., Nabb, D.L., Mingoia, R.T., Bischof, I., Nichols, J.W., Segner, H., Johanning, K., Han, X., 2015. Determination of metabolic stability using cryopreserved hepatocytes from rainbow trout (Oncorhynchus mykiss). Current Protocols in Toxicology 65, 4.42.41-44.42.29.

Franco, M.E., Schönenberger, R., Hollender, J., Schirmer, K., 2024. Organ-specific biotransformation in salmonids: insight into intrinsic enzyme activity and biotransformation of three micro-pollutants. Sci. Total Environ. 925, 171769.

Gomez, C.F., Constantine, L., Hugget, D.B., 2010. The influence of gill and liver metabolism on the predicted bioconcentration of three pharmaceuticals in fish. Chemosphere 81, 1189-1195.

Habig, W.H., Pubst, M.J., Jakoby, W., 1974. Glutathione S-transferases. The first enzymatic step in mercapturic acid formation. J. Biol. Chem. 249, 7130-7139.

Johanning, K., Hancock, G., Escher, B., Adekola, A., Bernhard, M.J., Cowan-Elsberry, C., Domoradzki, J., Dyer, S., Eickhoff, C., Embry, M., Erhardt, S., Fitzsimmons, P.N., Halder, M., Hill, J., Holden, D., Johnson, R., Rutishauser, S., Segner, H., Schultz, I., Nichols, J., 2012. Assessment of metabolic stability using the rainbow trout (Oncorhynchus mykiss) liver S9 fraction. Curr. Protoc. Toxicol. 53, 14.10.11-14.10.28.

Ladd, M.A., Fitzsimmons, P.N., Nichols, J.W., 2016. Optimization of a UDP-glucuronosyltransferase assay for trout liver S9 fractions: activity enhancement by alamethicin, a pore-forming peptide. Xenobiotica 46, 1066-1075.

Leitgeb, B., Szekeres, A., Manczinger, L., Vagvolgyi, C., Kredics, L., 2007. The history of alamethicin: a review of the most extensively studied peptaibol. Chem. Biodivers. 4, 1027-1051.

Lorenzen, A., Kennedy, S.W., 1993. A Fluorescence-Based Protein Assay for Use with a Microplate Reader. Analytical Biochemistry 214, 346-348.

McElroy, A.E., Kleinow, K.M., 1992. In-vitro metabolism of benzo [a] pyrene and benzo [a] pyrene-7, 8-dihydrodiol by liver and intestinal mucosa homogenates from the winter flounder (Pseudopleuronectes americanus). Mar. Environ. Res. 34, 279-285.

OECD, 2018. Test No. 319B: Determination of in vitro intrisic clearance using rainbow trout liver S9 sub-cellular fraction (RT-S9). OECD Guidelines for the Testing of Chemicals, Section 3 Paris: OECD Publishing.

Salminen, K.A., Leppänen, J., Venäläinen, J.I., Pasanen, M., Auriola, S., Juvonen, R.O., Raunio, H., 2011. Simple, direct, and informative method for the assessment of CYP2C19 enzyme inactivation kinetics. Drug Metab. Dispos. 39, 412-418.

Saunders, L.J., Fitzsimmons, P.N., Nichols, J.W., Gobas, F.A., 2020. In vitro-in vivo extrapolation of hepatic and gastrointestinal biotransformation rates of hydrophobic chemicals in rainbow trout. Aquat. Toxicol. 228, 105629.

Stresser, D.M., Blanchard, A.P., Turner, S.D., Erve, J.C.L., Dandeneau, A.A., Miller, V.P., Crespi, C.L., 2000. Substrate-Dependent Modulation of CYP3A4 Catalytic Activity: Analysis of 27 Test Compounds with Four Fluorometric Substrates. Drug Metabolism and Disposition 28, 1440-1448.
